# Ovarian Dropsy, Discharging Itself by the Umbilicus

**Published:** 1845-01

**Authors:** 


*Ovarian Dropsy, discharging itself by the Umbilicus.*—A woman, 28 years of age, had suffered for two years with dropsy of the right ovary. When the swelling had increased to a great size, the parts around the navel began to get hard; the navel itself projected, and finally gave way, upon which, and for a long time afterwards, there followed a discharge of purulent lymph, accompanied with a notable and progressive diminution of the abdominal enlargement. Shortly after her recovery this woman married, became pregnant, and gave birth to a strong boy. After delivery the abdomen did not shrink in the usual degree; by and by, on the contrary, it increased in size again, which increase was owing to an ovarian dropsy, which went on augmenting in size, and at length terminated in the formation of an abscess as on the former occasion.—*Ib., from Dr. Lambrecht, Medicinische Zeitung.*

